# Editorial: Bridging translational gaps in cardiovascular disease through large animal research

**DOI:** 10.3389/fcvm.2026.1824781

**Published:** 2026-03-23

**Authors:** Gerard Amorós-Figueras, André M. Leite-Moreira, Alessio Alogna, DeLisa Fairweather

**Affiliations:** 1 Department of Cardiology, Hospital de la Santa Creu I Sant Pau, Institut de Recerca Sant Pau (IR Sant Pau), CIBERCV, Universitat Autònoma de Barcelona, Barcelona, Spain; 2 RISE-Health, Department of Surgery and Physiology, Faculty of Medicine, University of Porto, Porto, Portugal; 3 São João Local Health Unit, Department of Anesthesiology, Porto, Portugal; 4 Department of Cardiology, Angiology and Intensive Care Medicine, Deutsches Herzzentrum der Charité (DHZC), Charité – Universitätsmedizin Berlin, Berlin, Germany; 5 German Centre for Cardiovascular Research (DZHK), Partner Site Berlin, Germany; 6 Department of Cardiovascular Medicine, Mayo Clinic, Jacksonville, FL, United States

**Keywords:** cardiovascular disease, experimental refinement, heart failure, large animal models, ovine, pulmonary hypertension, swine, translational research

Cardiovascular diseases (CVDs) remain the leading cause of global mortality, accounting for nearly 20 million deaths annually. As we enter an era defined by rapid advancements in *in silico* modeling, organ-on-a-chip technologies, and complex organoids, a critical question arises: Is the use of large animal models still relevant in modern cardiovascular research? While alternative models offer high-throughput potential and address specific ethical concerns, they often struggle to replicate the integrated systemic physiology electrophysiology and complex hemodynamics of the human heart. In this context, the use of large animal models in cardiovascular research has been fundamental in advancing our understanding of disease pathogenesis, facilitating the development of novel diagnostic tools and the validation of preventive and therapeutic strategies. This Research Topic, “*Bridging Translational Gaps in Cardiovascular Disease Through Large Animal Research*” brings together six novel studies that refine existing models, standardize diagnostic techniques, and evaluate novel therapeutic interventions.

The development of reliable disease models is the cornerstone of translational success. However, traditional models often suffer from high variability or excessive procedural stress. Berboth et al. address these limitations by presenting a refined porcine model of tachypacing-induced heart failure via a gradual increase in the rapid pacing frequency. Using jacketed external telemetry to monitor stress, the authors have demonstrated that stable dilated cardiomyopathy could be achieved with no significant alterations in cortisol levels or daily activity. This refined approach offers a more ethical and reliable platform for long-term therapeutic testing.

On the other hand, existing experimental models of chronic thromboembolic pulmonary hypertension (CTEPH) are often methodologically demanding and logistically impractical; furthermore, many fail to consistently reproduce severe pulmonary hypertension and right ventricular dysfunction, characteristic of the clinical phenotype. In this Research Topic, Moreira-Costa et al. present a straightforward swine model of severe CTEPH. Their new animal model utilizes repeated injections of silk sutures into the pulmonary arteries resulting in a phenotype that accurately mirrors human CTEPH, including consistently high mean pulmonary artery pressure, right ventricle (RV) hypertrophy, and altered gene expression of B-type natriuretic peptide and SERCA2a. This model provides a robust tool for investigating the mechanisms of RV failure and testing new pharmacological interventions.

As minimally invasive procedures like transcatheter aortic valve implantation (TAVI) become clinical standards, the need for large animal models to test device durability is paramount. Zhu et al. have described a standardized ovine aortic stenosis (AS) model through the surgical implantation of a novel bioengineered annular stent. This model effectively replicates the hemodynamic perturbations of clinical AS, such as increased transvalvular pressure gradients and peak flow velocity. Their study shows that TAVI achieved complete hemodynamic normalization in this model without accelerating bioprosthetic valve degeneration. This platform enables rigorous, long-term evaluation of TAVI performance and calcification progression in a setting that mimics human pathology.

Innovating in the treatment of vascular calcification, Liu et al. have performed a quantitative evaluation of intravascular lithotripsy (IVL), an adapted technology from ultrasound lithotripsy. Using a combination of *in vitro* gypsum models and *in vivo* swine studies, the authors have confirmed the efficiency of IVL in disrupting medial calcifications. The study also highlighted the safety of the procedure, demonstrating that IVL does not increase inflammatory status or significantly decrease the proportion of smooth muscle and endothelial cells in the treated artery. Such data are vital for confirming the biological safety of emerging adjunctive therapeutic modalities in managing severely calcified atheromatous lesions.

The untapped potential of neonatal tissues in cardiac repair represents a new frontier in regenerative medicine. Hughes et al. have explored the use of neonatally-derived multipotent stem cell clones to restore cardiac function. In a sheep model of myocardial infarction, allogeneic transplantation of these cells successfully restored cardiac function to normal levels, without the need for immunosuppression. This study is particularly significant for identifying the contribution of immunosuppressive mediators and paracrine effects that allow for stem cell retention and endothelial recruitment, paving the way for off-the-shelf allogeneic stem cell therapies.

Finally, the utility of many of these large animal models depends on the accuracy of our diagnostic tools. In this research topic, Billig et al. have proposed a method to perform standardized subxiphoid echocardiography (SE) in swine. The authors have described in detail the specific surgical approach and imaging protocol and also have provided reference values for parameters such as LV stroke volume, ejection fraction, and global longitudinal strain. Standardizing these measurements is essential for ensuring reproducibility across different research centers and improving the quality of translational cardiovascular data.

In conclusion, the six articles presented in this Research Topic reinforce that large animal models remain indispensable to translational medicine. Rather than being replaced by alternative methods, new technologies allow these models to be improved and more closely mimic clinical reality, thereby increasing their translational relevance. By bridging the gap between benchtop innovation and the bedside, these advancements ensure that large animal research remains more essential and rigorous than ever.

We would like to thank all the authors for their innovative submissions and the reviewers for their dedication to ensuring the quality of this Research Topic. We hope this collection serves as a valuable resource for scientists and clinicians working to bridge the gap in cardiovascular therapy.

